# Fixed point topology and robustness to perturbations between pairs of coupled neurons

**DOI:** 10.1186/1471-2202-13-S1-P174

**Published:** 2012-07-16

**Authors:** Sharon E Norman, Carmen C Canavier, Robert J Butera

**Affiliations:** 1 School of Electrical and Computer Engineering, Georgia Institute of Technology, Atlanta, GA 30332, USA; 2 Department of Cell Biology and Anatomy, Louisiana State University Health Science Center, New Orleans, LA 70112, USA; 3 Neuroscience Center of Excellence, Louisiana State University Health Science Center, New Orleans, LA 70112, USA; 4 Wallace H. Coulter Department of Biomedical Engineering, Georgia Institute of Technology, Atlanta, GA 30332, USA

## 

Synchronization of neural firing plays an important role in nervous system function. Synchronized and phase locked relationships have been implicated in the creation and retrieval of memories [1,2], while the disruption of normal synchrony and phase locking appears to be a factor in disorders like schizophrenia [3,4]. Here we explore synchronization mechanisms in small two-cell networks and the robustness of these networks to perturbations.

Phase response curves (PRCs) describe how neurons respond to perturbations applied at specific times during the interspike interval. The PRC can be plotted in terms of the time to next spike as a function of when the spike arrived; this curve describes the dynamics of a single neuron, and the intersection points of two of these interaction curves represent the fixed points of the coupled system [5,6].

Achuthan and colleagues have recently shown (manuscript submitted) that interaction curves with 0, 1, or 2 fixed points can exist in hybrid circuits of one biological and one model neuron. In this work, we use two reciprocally coupled Wang Buzsaki [7] model neurons to investigate how different numbers of coupled system fixed points affect the network robustness against perturbation. In addition, we investigate the effect of interaction curve shape on network robustness. The perturbation applied to test robustness is a delay or advance of a single spike of one model neuron.

Like Achuthan et al, we show that systems with 1 or 2 fixed points exhibit phase locking, while a system with 0 fixed points, but interaction curves in close proximity, shows temporary locking with some phase walkthrough. We are currently quantifying the effects of perturbation strength and timing on transient disturbances to the phase locking of the coupled system as well as convergence time back to the pre-perturbation phase relationship. In addition, we are exploring how the proximity of interaction curves affects robustness of the coupled system to perturbation. Preliminary results show that interaction curves that are closer together take a longer amount of time and more cycles to recover from perturbation than interaction curves that are further apart. Experimental validation of these modeling results will be shown.
